# Ten-year follow-up of sitagliptin treatment in patients with type 2 diabetes mellitus

**DOI:** 10.1186/s13098-021-00735-3

**Published:** 2021-10-24

**Authors:** Sachiko Hattori

**Affiliations:** Department of Endocrinology and Metabolism, Tohto Clinic, 4-1 Kioi-Cho, Chiyoda-Ku, Tokyo, 102-0094 Japan

**Keywords:** Dipeptidyl peptidase 4 inhibitor (DPP4i), Albuminuria-reducing effect, Islet-protective effect, Sitagliptin

## Abstract

**Background:**

Early and effective intervention with a dipeptidyl peptidase 4 inhibitor (DPP4i) before the development of advanced atherosclerosis in type 2 diabetes mellitus (T2DM) patients without a history of cardiovascular disease (CVD) is reported to increase the chance of significant reductions in not only microvascular disease, but also CVD.

**Method:**

This study aimed to investigate whether sitagliptin is effective and tolerated for glycemic control and whether renoprotective effects and β-cell function are preserved for as long as ten years in Japanese patients with T2DM without a history of CVD.

**Results:**

The situation is equivalent to improving glycemic control as assessed by hemoglobin A1c both in a sitagliptin group [sitagliptin 50 mg as either monotherapy or combination therapy with other oral glucose-lowering drugs (n = 17)] or a control group [placebo as either monotherapy or combination therapy with other glucose-lowering drugs (n = 9)], while anti-inflammatory effects as assessed by high-sensitivity C-reactive peptide in the sitagliptin group were superior to those in the control group. In the sitagliptin group, mean urinary albumin excretion (measured as urinary albumin-to-creatinine ratio) was markedly decreased, but no changes in estimated glomerular filtration rate were seen throughout the study. Beta-cell function as evaluated by homeostatic model assessment of β-cell function values was reduced at baseline in both groups, improved significantly in the sitagliptin group, and continued unchanged in the control group during the study.

**Conclusion:**

These observations suggest that early intervention with sitagliptin in patients with T2DM may have long-lasting renoprotective and islet-protective effects.

Trial registration: UMIN Clinical Registry (UMIN000038459). Registered 01 November (retrospectively registered): https://upload.umin.ac.jp/UMIN000038459

## Background

Sitagliptin was the first dipeptidyl peptidase 4 inhibitor (DPP4i) to receive marketing approval, and was launched in Japan at the end of 2009. Since then, sitagliptin has been shown to have a favorable therapeutic profile and is safe and effective for the majority of Japanese patients with type 2 diabetes mellitus (T2DM) for more than ten years. One year after the launch of sitagliptin in Japan, we reported that sitagliptin reduces albuminuria in patients with T2DM to show that a DPP4i could reduce albuminuria [1]. At that time, we recruited consecutive patients for a sample size of 60 subjects. We then followed-up the assigned patients who met all inclusion criteria and did not meet any exclusion criteria and were treated with or without sitagliptin for ten years. We show here that sitagliptin is effective and well tolerated for glycemic control and that renoprotective effects and beta-cell function were preserved for at least ten years in Japanese patients with T2DM.

## Methods

### Study design and participants

This single-center, open-label, randomized, prospective study included a total of 60 T2DM patients, 20–75 years old, with hemoglobin (Hb)A1c 8.0–6.5% and creatinine < 1.2 mg/dl regardless of diet, exercise, and medical treatment without a DPP4i for at least 12 months in this clinic. Patients were assigned at random to the sitagliptin group [sitagliptin (50 mg) as either monotherapy or combination therapy with other oral glucose-lowering drugs (n = 30)] or the control group [placebo as either monotherapy or other glucose-lowering drugs other than DPP4i (n = 22)]. Patients with type 1 diabetes, unstable cardiac disease, history of cardiovascular disease (CVD), significant renal impairment (creatinine clearance < 30 ml/min), or elevated (more than twice the upper limit of normal) alanine aminotransferase, aspartate aminotransferase, or creatine phosphokinase were excluded. All patients continued medication with oral glucose-lowering drugs (sulfonylureas, metformin, or pioglitazone) that had already been administered. All other medications including antihypertensives (angiotensin II receptor blockers or calcium channel blockers) and antihyperlipidemic agents (statins or fibrates), remained unchanged during the study. Patients were given detailed explanations of the study protocol, and informed consent was obtained from each patient. The study protocol was approved by the ethics committee of the Tohto Clinic. This trial was registered with the University Hospital Medical Information Network (UMIN000038459).

### Measurements and endpoints

For all patients, blood and urine samples were collected after overnight fasting at baseline and then at intervals of three months for ten years. Assessments of high-sensitivity C-reactive protein (hsCRP), immunoreactive insulin (IRI), and urinary albumin were performed at LSI Medicine Corporation (Tokyo, Japan). Other biochemical data were generated in house. Homeostatic model assessment of insulin resistance (HOMA-IR) was calculated as (fasting blood glucose (FBG) × IRI)/405. Homeostatic model assessment of β cell function (HOMA-β) was calculated as (IRI × 360)/(fasting blood glucose (FBG) -63). Data on adverse experiences, physical examinations, vital signs, electrocardiograms, and body weight were collected on each visit. All adverse experiences were rated by investigators for intensity and relationship to study drug. Laboratory evaluations included complete blood chemistry, hematology, and urinalysis.

The primary endpoint was change in urinary albumin excretion (measured as urinary albumin-to-creatinine ratio (ACR) in milligrams/gram of creatinine) and HOMA-β. Secondary endpoints were the occurrence of cardiorenal events, acute pancreatitis, and any cause of death for ten years.

### Statistical analysis

Data are expressed as mean ± standard deviation. Paired t-tests were used to compare parameters before treatment, and at every year after treatment. Statistical significance for differences was set at p < 0.05.

## Results

### Subjects

**Fig. 1:**
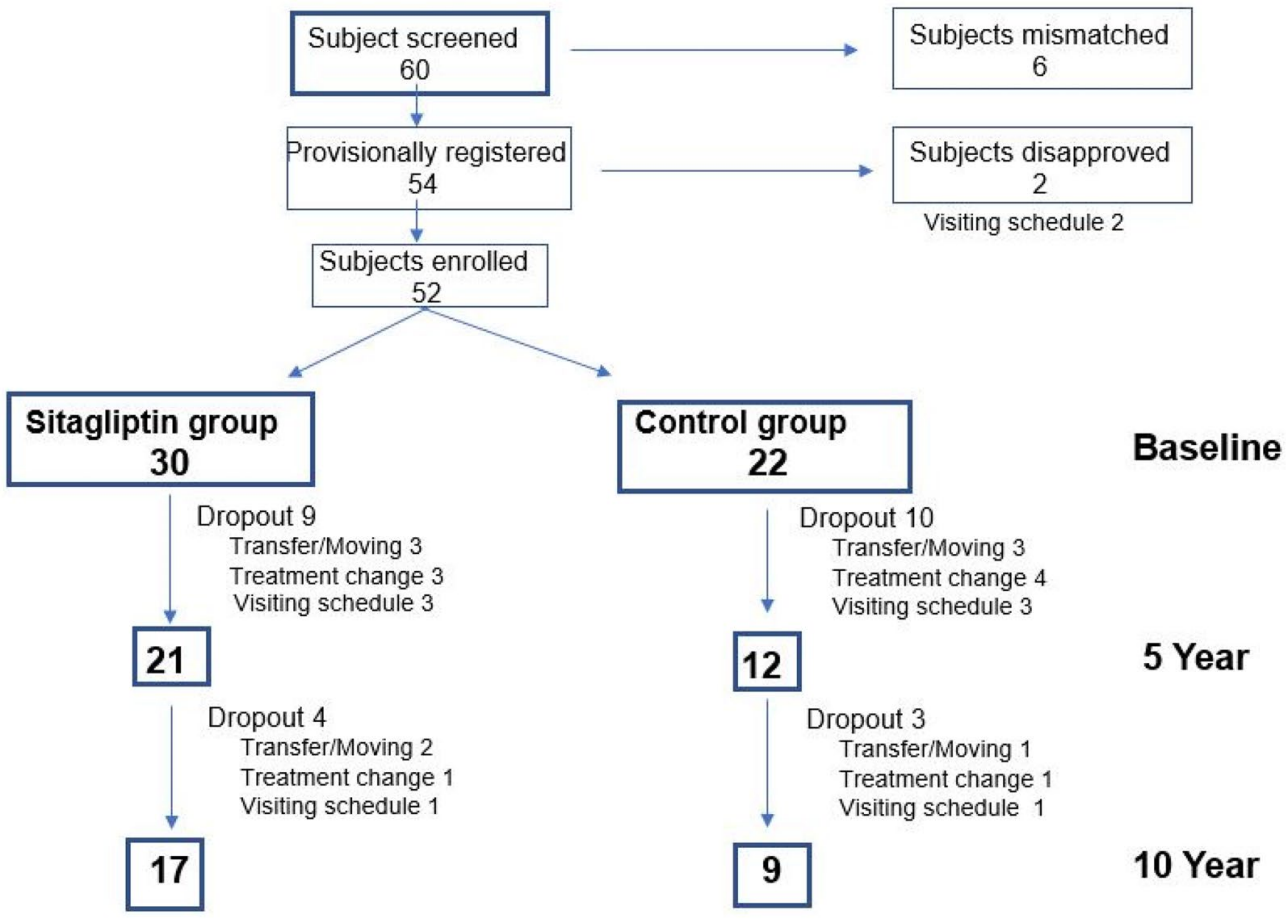
Progress of subjects through the study. In both the sitagliptin group and control group, all combination medications including oral glucose-lowering drugs and other medications (antihypertensives and antihyperlipidemic agents) remained unchanged throughout the entire duration of the study

We screened 60 patients, of whom 52 were included after applying the exclusion criteria (sitagliptin group: 30 vs control group: 22). Two patients declined to participate because of the visiting schedule. After 5 years, 19 subjects had dropped out (sitagliptin group: 21 vs. control group: 12). The final number of subjects who completed ten years of participation in the study was 26 (sitagliptin group: 17 vs control group: 9) (Fig. 1). The mean period from onset of T2DM to the start of this study was 9.1 ± 4.8 years in the final sitagliptin group, and 9.5 ± 6.7 years in the final control group. All combination medications including oral glucose-lowering drugs and other medications (antihypertensives and antihyperlipidemic agents) remained unchanged in both groups throughout the entirety of the study. With regard to oral glucose-lowering drugs, the final number of sitagliptin monotherapy was 5 in 17 patients, and that of placebo monotherapy number was 2 in 9 patients.

### Clinical parameters

In the sitagliptin group, no significant changes in body weight, BMI, or waist circumference were observed from the onset to 5 or 10 years of sitagliptin administration. Sitagliptin treatment for 6 months significantly lowered both systolic and diastolic blood pressures (SBP and DBP, respectively), an effect which continued through the study. A reduction in fasting blood glucose at 6 months (from 142 ± 38 to 130 ± 19 mg/dL), and a reduction in HbA1c at 6 months (from 6.9 ± 0.92 to 6.3 ± 0.70%) were observed, both effects of which were maintained through the study. Triglyceride, total cholesterol, HDL-cholesterol, and LDL-cholesterol were not altered by sitagliptin treatment (data not shown). Finally, a significant reduction in hsCRP from 452 ± 308 to 260 ± 131 ng/mL was observed at 6 months, and further decreases were observed throughout the study (Table 1).

**Table 1:** Clinical parameters in Sitagliptingroup and Control group

	Sitagliptin group (n = 17)							
Month of Study	0	6	12	24	48	72	96	120
FBG(mg/dl)	142 (38)	130 (19)*	136 (24)*	129 (22)*	128 (10)*	128 (12)*	112 (18)*	111 (14)*
HbA1c (%)	6.9 (0.92)	6.3 (0.70)*	6.6 (0.62)*	6.4(0.66)*	6.3 (0.42)*	6.3 (0.71)*	6.4 (0.20)*	6.3 (0.16)*
BMI	22.4 (3.4)	22.6 (3.3)	22.5 (3.1)	22.5 (3.7)	22.3 (3.2)	22.7 (3.2)	22.4 (3.6)	22.4 (3.7)
hsCRP(ng/ml)	452 (308)	260 (131)*	275 (189*)	190 (136)*	171 (139)*	160 (122)*	171 (77) *	162 (69)*
ACR(mg/gCr)	205 (390)	131 (263)	97 (286)	50 (165)	40 (102)	39 (109)	54 (130)	41 (105)
SBP (mmHg)	140 (18)	129 (19)*	129 (12)*	128 (14)*	128 (15)*	125 (13) *	124 (5)*	122 (4)*
DBP (mmHg)	78 (11)	73 (9)*	73 (8)*	71 (7)*	70 (10)*	69 (7)*	70 (6)*	69 (5)*

	Control group (n = 9)							
Month of Study	0	6	12	24	48	72	96	120
FBG(mg/dl)	137 (17)	129 (14)*	126 (17)*	119 (13)*	112 (15)*	121 (11)*	124 (9)*	124 (9)*
HbA1c (%)	6.8 (0.25)	6.3 (0.43)*	6.3 (0.35)*	6.3 (0.33)*	6.2 (0.44)*	6.2 (0.55) *	6.4 (0.27)*	6.4 (0.26)*
BMI	22.1 (3.3)	22.6 (3.3)	22.5 (3.1)	22.5 (3.7)	22.7 (3.9)	22.7 (3.2)	22.4 (2.9)	22.5 (3.0)
hsCRP(ng/ml)	204 (97)	241 (63)	291 (96)	287 (78)	269 (102)	266 (75)	220 (163)	240 (174)
ACR(mg/gCr)	99 (233)	103 (267)	125 (284)	129 (305)	132 (299)	132 (211)	115 (154)	119 (148)
SBP (mmHg)	130 (12)	130 (15)	131 (13)	131 (11)	130 (14)	129 (15)	130 (14)	131 (15)
DBP (mmHg)	72 (8)	72 (9)	73 (12)	73 (9)	72 (8)	73 (12)	73 (7)	72 (10)

Values are shown as means ± SD in parentheses

*FBG* fasting bloodglucose, *HbA1c* hemoglobin A1c, *BMI* body mass index, *hsCRP* high sensitivity C-reactitive protein, *ACR* albumin-to-creatinine ratio, *SBP* systolic blood pressure, *DBP* diastolic blood pressure

^*^Intragroup comparison: p < 0.05 (paired t test)

In the control group, no significant changes in body weight, BMI, or waist circumference were observed from the onset to 5 or 10 years of placebo administration. A reduction in fasting blood glucose at 6 months (from 137 ± 17 to 129 ± 14 mg/dL), and a reduction in HbA1c at 6 months (from 6.8 ± 0.25 to 6.3 ± 0.43%) were observed, both effects of which were maintained through the study. No significant changes in SBP, DBP, or hsCRP were observed during the study (Table 1).

### ACR and eGFR

**Fig. 2:**
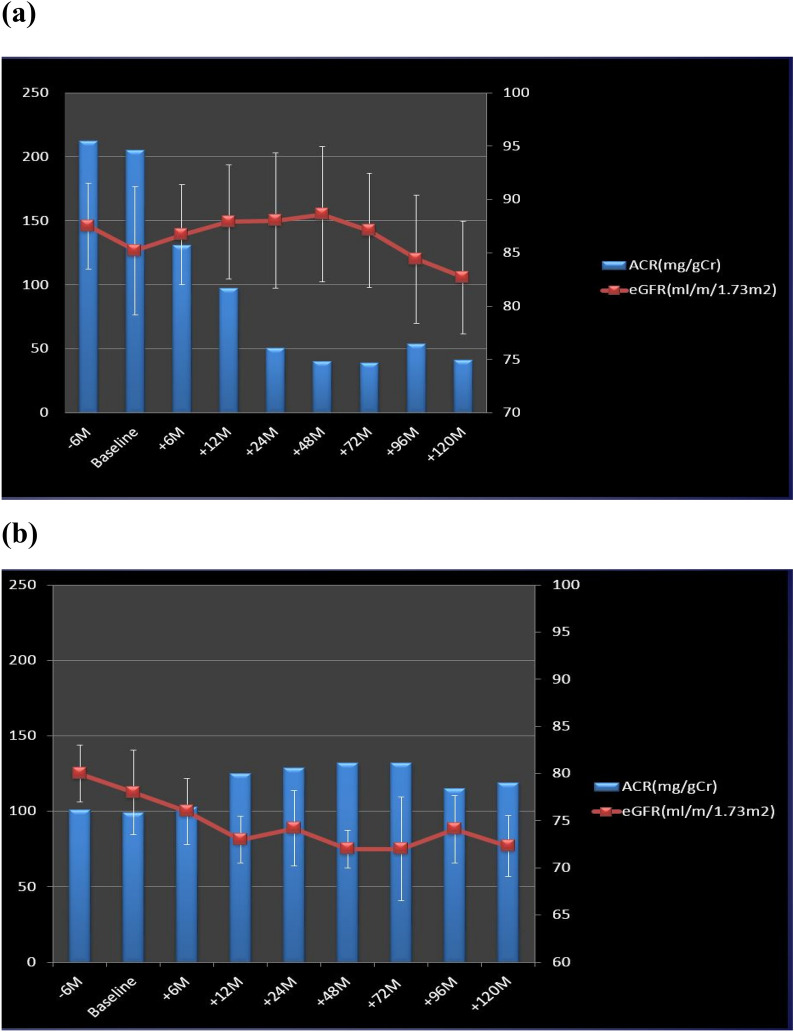
**a** Sitagliptin group. **b** Control group. Blue bars show mean ACR. Red squares show mean (± SD) eGFR

In the sitagliptin group, the mean urinary albumin excretion (measured as urinary albumin-to-creatinine ratio (ACR) in milligrams/gram of creatinine) did not change in the 6 months before sitagliptin treatment and markedly decreased in the 6 months after sitagliptin treatment, and these effects were gradually increased during the study. No changes in estimated glomerular filtration rate (eGFR) were seen throughout the study (Fig. 2a).

In the control group, mean ACR tended to slightly increase and eGFR tended to slightly decrease during the study (Fig. 2b).

Changes in mean ACR in both groups were not statistically significant because of the large standard deviations (SDs). Thus, Table 2 shows the staging for diabetic nephropathy by the Japanese Diabetes Society [2] before and after treatment with or without sitagliptin. The number of patients with improved stage was increased in the sitagliptin group, while the number of patients with a deterioration in stage increased in the control group.

**Table 2:** Staging of the participants for diabetic kidney disease

Staging for diabetic kidney diasese	Sitagliptin (n = 17)		Control (n = 9)	
Time (months)	0 M	120 M	0 M	120 M
Stage I	8	14	4	2
Stage II	7	2	4	6
Stage III	2	1	1	1
Stage IV	0	0	0	0
Stage V	0	0	0	0

Stage I: ACR < 30, Stage II: ACR30 ~ 300, Stage III: ACR > 300

*ACR* albumin creatinine ratio

### HOMA-β and HOMA-IR

In the sitagliptin group, HOMA-β values were reduced at baseline, significantly improved within 24 months, and then increased throughout the study. HOMA-IR values stayed within normal range during the study (Table 3). In the control group, HOMA-β values, which were similarly reduced at baseline, continued unchanged and HOMA-IR values also remained within normal range during the study (Table 3).

**Table 3:** HOMA-β and HOMA-IR in Sitagliptin group and Control group

	Sitagliptin group (n = 17)							
Month of Study	0	6	12	24	48	72	96	120
HOMA-β	15.6 (8.81)	13.1 (8.15)	19.5 (10.5)	20.3 (7.41)*	24.6 (8.60)*	28.12 (8.91)*	31.2 (12.2)*	31.4 (10.41)*
HOMA-IR	1.15 (0.74)	1.14 (0.63)	1.14 (0.60)	1.12 (0.59)	1.15 (0.61)	1.18 (0.55)	1.14 (0.61)	1.15 (0.54)

	Control group (n = 9)							
Month of Study	0	6	12	24	48	72	96	120
HOMA-β	21.6 (18.7)	22.0 (16.6)	19.5 (13.1)	22.4 (18.7)	20.9 (21.2)	21.1 (4.2)	24.2 (18.5)	23.2 (18.4)
HOMA-IR	1.14 (0.81)	1.36 (0.67)	1.27 (0.61)	1.41 (0.74)	1.29 (0.76)	1.21 (0.66)	1.22 (0.52)	1.17 (0.50)

Values are shown as means ± SD in parentheses

*HOMA-β*, homeostatic model assessment of β cell function, *HOMA-IR* homeostatic model assessment of insulin resistance

^*^Intragroup comparison: p < 0.05 (paired t test)

### Safety and tolerability

Starting with 52 subjects, the final number of subjects after ten years was 26. Although half of the subjects dropped out, no episodes of hypoglycemia exhibiting marked severity (i.e., loss of consciousness or requirement for medical assistance), no heart failure and no acute pancreatitis was observed during the study in either group.

## Discussion

The major findings for sitagliptin in this study were a persistent albuminuria-reducing effect and islet-protective effect developing over a period of years.

In one year after the launch of sitagliptin in Japan, we reported that sitagliptin reduced albuminuria without changing eGFR in patients with T2DM [1]. That was the first report in the world to show that sitagliptin could reduce albuminuria. Here, we showed that this effect of sitagliptin continued for ten years, while albuminuria tended to increase somewhat without sitagliptin despite reasonably good glycemic control in both groups. On the other hand, sitagliptin showed a small but early decline in eGFR that lasted for 48 months with no difference in changes of ACR in the Trial Evaluating Cardiovascular Outcomes with Sitagliptin (TECOS) trial [3, 4].

Likewise, early and effective intervention with a DPP4i before the development of advanced atherosclerosis in T2DM patients without a history of CVD is reported to increase the chance of a significant reduction not only of microvascular disease, but also CVD [5, 6], while three large-scale randomized clinical trials including TECOS have reported that DPP4i did not reduce CV risk in T2DM patients with a history of CVD or at high risk of CVD [7–9].

Thus, both renoprotective and anti-atherosclerotic effects might be expected with earlier use of sitagliptin.

Ascener et al. reported that sitagliptin (100 or 200 mg) as monotherapy for 24 weeks significantly improved HOMA-β (100 mg sitagliptin: from 57 to 70%; 200 mg sitagliptin: from 55 to 68%), while placebo did not [10]. We also showed here that sitagliptin (50 mg) as either monotherapy or in combination therapy with other oral glucose-lowering drugs for ten years significantly improve HOMA-β. Although HOMA-β values at baseline was lower and doses of sitagliptin were smaller in our study compared with the investigation by Ascener et al., the effects of sitagliptin in improving HOMA-β values were similarly clear within 24 months, although to a lesser extent, then gradually increasing until the end of our study. Malvandi et al. reported that sitagliptin effectively modulated beneficial immune-relevant pathways in a human beta cell line. This might be related to the islet-protective effects seen in T2DM patients treated with sitagliptin [11].

East Asian T2DM is becoming widely recognized as characterized primary by β-cell dysfunction and generally lesser obesity and higher insulin sensitivity compared with that in Caucasians [12–15]. Indeed, HOMA- β values were decreased and HOMA-IR values were within normal limits at the start of this study, while HOMA-β remained more than 50% at baseline in the study by Ascener et al. [10].

However, UKPDS data have shown that decreased insulin sensitivity is a prerequisite for T2DM development and β-cell function is reduced to about 50% at the diagnosis of type 2 diabetes, with a 4% rate of reduction per year among Caucasians [15, 16]. Thus, the islet-protective effect of sitagliptin seems likely to prove less effective once T2DM has developed among individuals from a Caucasian background despite the data reported by Ascener et al. [10].

Participants in both the sitagliptin group (n = 17) and the control group (n = 9) completed this study receiving counseling on adequate exercise and diet throughout the study. Probably for these reasons, the situation is equivalent to improving glycemic control in both groups, while anti-inflammatory effects in the sitagliptin group were superior to those in the control group. Thus, the persisting albuminuria-reducing and islet-protective effects of sitagliptin might be related to anti-inflammatory effects and/or other unknown factors, rather than through glucose-lowering effects. Interestingly, Solerte et al. reported that sitagliptin treatment was associated with reduced mortality and improved clinical outcomes in a cohort of patients with T2DM and coronavirus disease 2019 (COVID-19) at the time of hospitalization [17, 18]. As severe acute respiratory syndrome coronavirus 2 (SARS-CoV-2) may bind to DPP4 (or CD26), which mediates proinflammatory signals, sitagliptin might exert anti-inflammatory effects in patients with T2DM and COVID-19.

The key limitation of the present study was the small number of participants. Ten years of follow-up without any change in treatment obviously represents a substantial barrier to participation, and many participants indeed dropped out during the study. Nevertheless, this observation for ten years suggests that early intervention with sitagliptin might have long-term clinical significance.

## Conclusion

This study shows the good overall safety profile of sitagliptin, as well as its efficacy in improving glycemic control over the long term. Also, this study suggests that sitagliptin may have long-lasting renoprotective and islet-protective effects in patients with T2DM.

## Data Availability

The datasets analyzed during the current study are not publicly available due to some relevant ongoing studies, but may be available from the corresponding author of this article on reasonable request.

## Abbreviations

ACR: Albumin-to-creatinine ratio
BMI: Body mass index
CVD: Cardiovascular disease
DBP: Diastolic blood pressure
DPP4: Dipeptidyl peptidase 4
FBG: Fasting blood glucose
eGFR: Estimated glomerular filtration rate
HbA1c: Hemoglobin A1c
HDL: High-density lipoprotein
HOMA-β: Homeostatic model assessment of β-cell function
HOMA-IR: Homeostatic model assessment of insulin resistance
IRI: Immunoreactive insulin
LDL: Low-density lipoprotein
hsCRP: High-sensitivity C-reactive protein
SBP: Systolic blood pressure
T2DM: Type 2 diabetes mellitus
